# The relationship between perceived educational opportunity fairness and social trust: an empirical study based on cognition, emotion and developmental expectation

**DOI:** 10.3389/fpsyg.2026.1876898

**Published:** 2026-08-11

**Authors:** Huijuan Hou, Ruqiao Wu, Dongliang Wang

**Affiliations:** School of Law, Chongqing University, Chongqing, China

**Keywords:** perceived educational opportunity fairness, social fairness, social mobility expectation, social trust, subjective well-being

## Abstract

The relationship between perceived educational opportunity fairness and social trust is a crucial issue concerning social stability and institutional identification. However, existing research has predominantly focused on the direct relationship of educational attainment, with few studies providing a granular analysis of the complex psychological mechanisms through which fairness perceptions are transformed into social trust. Utilizing data from the 2023 Chinese General Social Survey (CGSS), this study employs Ordered Logit regression, Ordinary Least Squares (OLS), and stepwise regression to construct an integrated multiple mediation model encompassing cognition, emotion, and expectation. Furthermore, the moderating role of internet usage intensity in this process is examined. The findings indicate that: (1) Perceived educational opportunity fairness significantly predicts higher levels of social trust, a conclusion that remains robust across multiple sensitivity tests; (2) Mediation analysis reveals that educational opportunity fairness indirectly influences social trust through three distinct pathways: sense of social fairness (cognitive), subjective well-being (emotional), and subjective social mobility expectations (developmental expectation); (3) Moderation analysis shows that internet usage intensity significantly weakens the positive association between educational opportunity fairness and social trust. Specifically, the higher the intensity of internet use, the weaker the positive effect of fairness on trust; (4) Overall, the relationship between educational opportunity fairness and social trust is characterized by a multi-path mechanism of “cognition-emotion-developmental expectation.” This paper reveals the social and psychological contexts underlying the links between educational opportunity fairness and social trust. It suggests that while optimizing the allocation of educational resources, policymakers should also refine the mechanisms for guaranteeing fair educational opportunities. In the pursuit of balanced resource allocation, emphasis should be placed not only on objective inputs but also on ensuring the transparency and justice of distribution procedures, thereby eliminating the interference of non-effort factors such as household registration (hukou) and family background.

## 1 Introduction

In both traditional and modern Chinese cultural values, equitable access to educational resources serves as the “barometer” of societal values. It precisely gauges a society’s understanding of justice, dignity, and opportunity, while functioning as a cornerstone for social equality and a vital guarantee for national prosperity (Chen and Xiang, 2021). Following the basic popularization of education, the equity of resource acquisition has emerged as a critical issue for social development (Chen, 2006). Equitable access to educational resources fosters a collaborative and trusting relationship between the state and society: when children from different social strata grow up together in a relatively fair environment, they perceive society as orderly and merit-based. This cultivates a robust sense of citizenship and trust in public institutions (Ishimaru et al., 2018). Conversely, severe inequality in resource acquisition breeds fracture and resentment: if education becomes an “accelerator” of class solidification, disadvantaged children are deterred by their lack of access to high-quality resources. Consequently, the “social lesson” they internalize is that rules are arbitrarily rewritten by capital and power. This ultimately fragments societal values-leading some to believe in personal agency and effort, while others succumb to the cynicism of intergenerational privilege, eroding social cohesion and stability (Guillen and Zeichner, 2018). Crucially, the perception of educational equity spans beyond the mere equitable distribution of institutional resources; it is deeply rooted in the subjective fulfillment of relational needs within learning interactions. Regarding the need for relatedness, a sense of educational equity stands as a primary indicator of an individual’s belongingness and security within the educational environment. When individuals perceive that educational resources are reasonably allocated, algorithmic feedback is fair, and learning opportunities are equal, their social relatedness needs are fully satisfied (Zeichner et al., 2016). In terms of the need for competence, the perception of educational equity serves as a vital psychological resource that fosters self-efficacy during educational attainment. It constitutes the psychological cornerstone for mutual understanding among social members and the formation of a shared value identity. This psychological asset not only empowers individuals to utilize knowledge to overcome difficulties, regulate emotions, and navigate interpersonal relationships, but also provides free or low-cost pathways for psychological growth across different income brackets, occupations, and age groups, thereby enhancing public mental health literacy from its root (Yu, 2026). There is a close relationship between cognition, emotion and social trust: social individuals with higher cognitive ability and better emotional understanding ability have more pro-social psychological attitudes and behaviors (Shang and Su, 2020). The transformation of social justice into social trust in educational equity is a complex psychological process in which social individuals start from rational cognition and finally point to positive future expectations through the catalysis of emotional resonance (Allan and Jacquelynne, 2000). Each step of this process is closely linked to the beliefs and values held by community residents (Mayhew and Fernández, 2007). In particular, when community residents feel recognized and valued by the social system through educational initiatives, their abstract cognition of social justice materializes into a nurturing ground for institutional trust, ultimately translating the perception of societal fairness into systemic trust in educational justice (Vetter et al., 2022).

In recent years, the Chinese government has consistently increased its investment in education and supported substantial expansion in the supply of educational resources. According to the Statistical Communiqué on the Execution of National Education Funds in 2024, jointly released by the Ministry of Education, the National Bureau of Statistics, and the Ministry of Finance, the total national investment in education funds reached 6,889.924 billion CNY in 2024, representing a 6.66% increase over the previous year. Notably, national fiscal education expenditures have remained stable at over 4% of the Gross Domestic Product (GDP) for 13 consecutive years, reaching 4.02% in 2024. This trend indicates that the Chinese education system has established a relatively stable institutional guarantee for funding at the macro-level. However, in contrast to this robust macro-level investment, significant variations persist in individuals’ subjective perceptions of educational opportunity fairness at the micro-level. Among the myriad factors influencing social trust, education opportunity equity and education resource acquisition equity are widely regarded as one of the two critical predictors (Österman, 2021). Education is not only linked to heightened cognitive abilities and social participation, but also corresponded to the cultivation of social norms, thereby exhibiting a positive relationship with social trust (Cary, 2021). At the individual level, people with higher educational attainment typically enjoy superior employment opportunities and income levels, while residing in environments characterized by more stable social norms and lower crime rates, thereby accumulating more abundant economic and social resources. These resource advantages not only bolster personal sense of security and social participation capacity but also enhance individuals’ confidence in assuming potential risks when interacting with strangers, which frequently relates to higher levels of social trust (Huang et al., 2009). Conversely, for individuals with lower educational attainment, disadvantaged economic conditions and social status tend to restrict their social interactions, further impeding the cultivation of social trust (Gao and Zhao, 2022). However, this explanatory framework emphasizes the economic and social advantages brought by the level of education itself, while relatively overlooking the potential link between the perception of educational fairness and social trust.

Social trust does not emerge in isolation; rather, it is shaped by a multitude of factors, including socio-economic conditions, the institutional environment, and social norms (Liu and Wei, 2025). Among these factors, fairness is a crucial construct associated with social trust. Fairness serves to reduce social distance and bridge social gaps between individuals, thereby corresponding to a stronger bond of trust among members of society (Cho, 2016). When significant inequality or social polarization exists within a society, the level of trust among its members tends to decline. For instance, a substantial body of research has identified a significant negative correlation between income inequality and social trust (Albertazzi et al., 2025). An unequal social environment tends to foster social fragmentation and status competition, thereby undermining social cohesion and the foundation of trust. As economic inequality is closely linked with societal division, social trust is increasingly placed at risk (Kanitsar, 2022).

Existing researches indicate that people obey the law because they identify with the legitimacy of the system (Tyler, 2006). In the realm of education, the process of allocating educational opportunities functions, in essence, as a mechanism of institutional selection. Education level is an important factor in reducing various forms of social inequality and ensuring that the general public has access to better health services (Akokuwebe and Idemudia, 2022). When individuals perceive educational opportunities as equitable, they develop a sense of identification with the educational system, which often manifests as a positive predictor of social trust. As socioeconomic development continues to advance, public demand for high-quality educational resources and equitable opportunities grows steadily; consequently, the unequal distribution of such opportunities shares a potential link with how members of society evaluate the fairness of their social institutions. Concurrently, the role of fairness in the formation of social trust has garnered widespread attention. On the one hand, existing researches indicate that individuals’ subjective perceptions-and the shifts therein-regarding their own economic status and social standing constitute a primary pathway through which inequality of opportunity influences social trust (Yang et al., 2025). People generally find it easier to accept inequality in outcomes resulting from differences in effort, whereas they are more sensitive to-and react more negatively toward-inequality of opportunity (He et al., 2022). On the other hand, existing researches indicate that the perception of social fairness is a significant factor associated with social trust; individuals who perceive society as a whole to be fair are more likely to develop higher levels of social trust (Li and He, 2022). If members of society perceive educational opportunities as equitable, they are more inclined to believe that social norms are just, thereby strengthening their trust in social institutions and other individuals. Conversely, when access to education is linked with background barriers such as family background, the household registration system, or the allocation of social resources, educational inequality erodes members’ trust in social institutions. Furthermore, a positive predictive relationship is observed between subjective well-being and social trust; specifically, social trust is a positive predictor of individual happiness, sharing close links with social relationships, health status, and perceptions of social status (Lu et al., 2020). Social stratification and opportunities for social mobility also shape an individual’s life chances and social cognition, thereby relating closely to their level of social trust (Akaeda, 2022).

Based on the above analysis, this paper utilizes data from the 2023 CGSS to examine the impact of perceived educational opportunity fairness on social trust and the underlying mechanisms through which this effect operates. The research contributions of this study are primarily reflected in four aspects. Firstly, adopting the perspective of individual perception, this paper elucidates the mechanisms through which perceived educational opportunity fairness shapes social trust. Secondly, by constructing a multiple mediation model-encompassing the perception of social fairness (cognition), subjective well-being (emotion) and expectations regarding social mobility (developmental expectation), this study systematically analyzes the psychological logic underpinning the formation of social trust. Thirdly, this paper investigates the moderating role of internet usage within the aforementioned relationships, thereby providing new empirical evidence for social governance and educational equity in the digital era. Fourthly, this study integrates three distinct psychological dimensions as mediators: subjective well-being at the emotional level, social perception at the cognitive level, and social mobility expectations. By doing so, it elucidates the underlying mechanisms through which perceived educational opportunity fairness shapes and translates into social trust.

## 2 Literature review and research hypotheses

### 2.1 Equality of educational opportunity and social trust

Equality of educational opportunity stands as a paramount principle in the field of education and serves as a crucial metric for assessing distributive justice within society. Although academic discourse regarding its conceptual definition encompasses a multifaceted array of considerations-including the allocation of educational resources, the socioeconomic backgrounds of students, and the internal environment of schools -its essence fundamentally centers on whether an individual’s selection within the educational system is subject to interference from factors unrelated to their own effort (Coleman, 1975). In economic research, perceived educational opportunity fairness is typically subsumed under the theory of “equality of opportunity” (Dworkin, 1981). This theory emphasizes that an individual’s social outcomes are typically modeled as a function of two categories of factors: personal effort, and external environmental factors beyond the individual’s control-such as family background, region of birth, and parental education level (Checchi and Peragine, 2010). According to this theory, an individual’s success should depend on their own efforts, rather than on uncontrollable external circumstances-such as family background, economic conditions, or differences in household registration (Van de Gaer and Ramos, 2020). When disparities in educational attainment stem primarily from ascribed factors rather than individual effort (Golley and Kong, 2018), they constitute a grave inequality of opportunity-an inequality that frequently corresponds to the persistent self-reproduction of social class through differences in cultural capital (Bourdieu and Passeron, 1990). In recent years, scholars have begun to explore how such inequality relates to individuals’ social attitudes-particularly its role associated with social trust. As a generalized psychological expectation, social trust is closely tied to an individual’s assessment of the legitimacy of the rules governing the functioning of society (Tyler, 2006). Perceived educational opportunity fairness highly relevant to whether people perceive social institutions to be implemented fairly. On one hand, higher levels of education typically correspond to broader’s cognitive abilities, social engagement, and economic resources, thereby serving as positive predictors for social trust (Huang et al., 2009). On the other hand, however, if the educational system were to devolve into a mechanism for entrenching social stratification, the associated negative implications for societal cohesion could be substantial. Yang et al. (2025) confirms that the public is more sensitive to inequality in educational opportunities than to inequality in outcomes; specifically, when access to education is constrained by factors such as family background, disadvantaged groups-including those with low incomes, limited education are more prone to experiencing a profound sense of injustice, which in turn relates negatively to their trust in both social institutions and society at large.

The aforementioned pathways regarding the perception of fairness and the construction of trust reflect the process by which individuals identify with the educational system. When the distribution of educational opportunities tends toward fairness, individuals are more inclined to believe that social rules rewards those who are diligent and capable, rather than favoring those with privileged backgrounds. This perception of institutional fairness becomes internalized as stable psychological capital, which statistically predicts a higher propensity for trust among members of society. Based on this premise, the present study posits that perceived fairness in educational opportunities serves as a vital foundation closely linked with enhanced social trust and mitigated social divides. Accordingly, this paper proposes the following research hypotheses:

Hypothesis 1: Residents’ perceived level of educational opportunity fairness is positively associated with, and serves as a significant positive predictor of, their level of social trust.

### 2.2 The mediating role of perceived social fairness

The manner in which educational opportunities are distributed constitutes not only a core domain of social resource allocation but also relates closely to individuals’ evaluations of the overall fairness of society. In the study of the mechanisms underlying the formation of social trust, scholars have gradually shifted their focus from the macro-level institutional environment to micro-level psychological cognition. Within this context, the sense of social fairness-defined as an individual’s subjective evaluation of the legitimacy of social resource distribution and institutional functioning-not only reflects their perception of institutional legitimacy but also constitutes a value judgment regarding the overall functioning of the social environment (Sui et al., 2024). Research indicates that a sense of social fairness serves as a key psychological mediator linking objective social structures to subjective behavioral choices. This sense of social fairness is typically categorized into the perception of procedural fairness (fairness of opportunity) and the perception of distributive fairness (fairness of outcome). Specifically, procedural fairness primarily reflects an individual’s assessment of whether social members have equal access to opportunities for development, whereas distributive fairness focuses more on whether the ultimate allocation of social resources is equitable (Hou et al., 2023). From the perspective of institutional analysis, an equitable institutional environment serves as a crucial institutional foundation associated with higher levels of social trust. Cross-national empirical studies indicate that, compared to factors of social homogeneity, institutional fairness possesses greater explanatory power regarding social trust. When social institutions provide fair procedural rules, equitable administration, and a fair distribution of income, individuals are more inclined to form positive evaluations of these institutions and, consequently, exhibit higher levels of social trust (You, 2012). However, objective institutions do not directly translate into social trust; rather, their efficacy is often realized through individuals’ subjective internalization of the institutions’ fairness. In other words, when individuals believe that social institutions adhere to fair procedural rules-particularly regarding the allocation of resources and opportunities-this sense of procedural justice is robustly linked to the expectation that other members of society will abide by those same rules, thereby exhibiting a positive predictive relationship with their level of social trust.

Lamertz points out that individuals often evaluate whether institutions are fair-by engaging in social comparison and drawing upon social information and social networks-thereby forming an overall perception of society (Lamertz, 2002). In social interactions, individuals commonly exhibit an aversion to unfair distribution-a phenomenon known as “fairness preference.” When members of a society perceive that resources are being distributed unfairly, their willingness to cooperate and their levels of trust often decline (Fershtman et al., 2012). Given that education bears upon intergenerational mobility as well as the distribution of income levels and social status, the fairness of educational opportunities within this process is tightly bound up with how individuals interpret the integrity of the social contract. Based on the aforementioned logic, this study posits that the perception of educational opportunity fairness serves as a vital antecedent in predicting an individual’s overall sense of social fairness. When the distribution of educational opportunities is perceived as more equitable, individuals are more inclined to view social institutions as rational and legitimate, thereby exhibiting a heightened sense of social fairness; conversely, when access to educational opportunities appears significantly influenced by factors such as family background or social environment, individuals perceive an inequity in the allocation of social resources, which systematically corresponds to lower levels of social trust in both other individuals and institutions. Accordingly, this paper proposes the following research hypotheses:

Hypothesis 2: Perceived educational opportunity fairness is indirectly and positively associated with individuals’ levels of social trust, a relationship that is mediated by their sense of social fairness.

### 2.3 The mediating role of subjective well-being

Subjective well-being is an individual’s overall evaluation of their own life circumstances; it serves as a key indicator of personal quality of life and typically encompasses assessments of life satisfaction, as well as positive and negative emotions (Diener, 1984). In social psychological research, subjective well-being is not merely a matter of individual perception, but exhibits close links with social relationships and the social environment (Lu et al., 2020). Educational equity has been proved to be one of the significant predictors of life satisfaction, and the degree of educational support is considered to be an important factor affecting the life quality of social individuals (Akokuwebe et al., 2025). In recent years, an increasing number of scholars have focused on how socio-environmental factors relate to individual psychological well-being. Among these factors, subjective well-being serves not only as a key indicator for evaluating an individual’s quality of life but also as a psychological mechanism bridging the perception of institutional fairness and social attitudes. Indeed, socio-situational factors-such as distributive injustice and the denigration of social status-are often linked to lower levels of individuals’ mental health. For instance, in a study examining the subjective well-being of ethnic minorities within the context of social transition, Kus et al. (2014) found that social inequality, as well as individuals’ perceptions regarding the legitimacy of their social status, significantly influenced their life satisfaction. When individuals perceive the existence of unfairness or social status denigration within society, their sense of self-esteem and security is challenged, which systematically corresponds to a marked decline in their life satisfaction (Kus et al., 2014). Furthermore, social engagement and social interaction are likewise considered crucial avenues associated with higher personal well-being. At their core, they serve to strengthen the psychological bond between the individual and society (Albanesi et al., 2007).

A higher level of subjective well-being statistically predicts a greater propensity for social trust in others. When stable trust relationships are established among members of society, social interactions become smoother, reflecting a more positive social environment for individuals (Flores and Solomon, 1998). There exists a significant positive correlation between social trust and subjective well-being; individuals with high levels of well-being typically hold more positive evaluations of their social environment and are more inclined to believe in the goodwill of others. Furthermore, the establishment of trusting relationships serves to reduce the perception of risk in social interactions (Rahn and Transue, 1998), thereby aligning with a symbiotic, multi-pathway configuration of “well-being-trust-social reciprocity.”

In modern society, education is regarded as a crucial avenue for achieving social mobility and enhancing life opportunities. Consequently, the fairness of educational opportunities not only shapes individuals’ evaluations of social institutions but also relates closely to their overall assessment of their own life circumstances. The fairness of educational opportunities carries heightened symbolic significance regarding social mobility, making it more likely to influence individuals’ judgments concerning their future developmental prospects (Breen and Jonsson, 2005). Perceived equality of educational opportunity exhibits a positive predictive relationship with individuals’ psychological well-being and their life satisfaction; this positive emotional state, in turn, serves as a robust positive predictor of generalized trust toward others. In other words, subjective well-being serves as a crucial emotional mediator in the process whereby the perception of institutional fairness aligns with positive social attitudes. Accordingly, this paper proposes the following research hypotheses:

Hypothesis 3: Perceived educational opportunity fairness is indirectly and positively associated with individuals’ social trust through a mediating pathway characterized by heightened subjective well-being.

### 2.4 The mediating role of social class mobility

Social class mobility is typically viewed as the process by which an individual transcends their original social stratum within a system of social stratification, thereby achieving a shift in status. In contrast to objective levels of social mobility, a psychological perspective places greater emphasis on individuals’ expectations regarding social mobility-specifically, their perceptions and anticipations regarding the likelihood that they and their descendants can achieve upward mobility through effort (Day and Fiske, 2017). When individuals perceive opportunities for social mobility as limited, their social attitudes tend to become negative, and their trust in social institutions and fellow members of society diminishes. In the study of social inequality, subjective cognitive factors are increasingly supplanting objective indicators as the central variables for predicting psychological responses. Merten and Niedringhaus (2025) points out that studies on economic inequality have long focused on objective indicators while neglecting individuals’ subjective perceptions of inequality and social mobility opportunities. Nevertheless, an individual’s perception of social mobility opportunities significantly associated with their evaluation of the social environment, as well as various psychosocial outcomes such as political attitudes. From an empirical point of view, individuals’ perceptions of economic inequality co-vary with their levels of social status anxiety, which exhibites a close link with their expectations regarding opportunities for upward mobility. When individuals perceive that opportunities for upward mobility are diminishing, social inequality is more likely to be accompanied by feelings of anxiety and dissatisfaction (Melita et al., 2024). This study indicates that the anxiety stemming from negative expectations regarding opportunities for social mobility serves as a deep-seated psychological predictor related to the downward shift in the willingness to engage in social cooperation and of interpersonal trust. Equitable opportunities for social mobility are robustly linked to the foundation of social trust. When members of society believe that they can achieve upward social mobility through their own efforts, they are more inclined to form positive assessments regarding the fairness of social institutions, which systematically corresponds to higher levels of social trust both in the operational rules of society and their fellow human beings (You, 2012). This “possibility of upward mobility” serves as a “social glue” for social governance, effectively mitigating the risk of social exclusion and fostering individuals’ active integration into social networks and the establishment of reciprocal relationships (Stanley et al., 2011).

Educational opportunities play a pivotal role in the process of social mobility. Education is widely regarded as a crucial avenue for upward social mobility; when educational opportunities are more equitable, individuals are more inclined to believe that they can elevate their social status through hard work, thereby exhibiting a higher expectation of social mobility. Conversely, when access to education is constrained by family background or other social factors, individuals tend to perceive the channels for social mobility as obstructed, which in turn erodes their trust in social institutions. Consequently, perceived fairness in educational opportunities is not only directly associated with social trust, but it also shares an indirect link by predicting individuals’ expectations of their own social class mobility. Accordingly, this paper proposes the following research hypotheses:

Hypothesis 4: Perceived educational opportunity fairness is indirectly and positively associated with individuals’ social trust through a mediating pathway characterized by heightened expectations of social class mobility.

### 2.5 The mediating role of internet usage

With the pervasive penetration of digital technologies, the Internet has evolved from being merely a tool for information transmission into a pivotal instrument for closely associated with the variation in individuals’ social cognition. Existing research indicates that media and information dissemination platforms play a critical role in shaping individuals’ social perceptions and their evaluations of institutions. People frequently rely on media to acquire information regarding the functioning of society, and subsequently adjust their attitudes toward social institutions and political systems based on this information (Cheng, 2020). You et al. (2022) utilized data from the China Family Panel Studies (CFPS) and found that-compared to those who do not use the internet-individuals who do use it exhibit lower levels of political trust. The study points out that the internet usage corresponds to shifts in public evaluations of public institutions, which is tied to the diversification of channels for accessing information and a heightened awareness of social issues (You et al., 2022). The decentralized and fragmented nature of online information makes it easier for individuals to encounter negative information-such as social conflicts-which frequently relates negatively to their trust in public institutions and members of society.

The internet environment has vastly expanded the scope of social comparison and intensified individuals’ perceptions of social inequality. An empirical study of psychology indicated that individuals’ perceptions of economic inequality depend not only on objective economic disparities but also covary with social relationships and the information environment (Willis et al., 2024). As individuals engage with a wider array of diverse social groups within the internet environment, they become more acutely aware of disparities in the allocation of social resources, thereby intensifying their perception of social inequality. In the realm of education, the internet not only disseminates specific instances of the unequal distribution of high-quality resources but also amplifies the advantages conferred by privilege in educational selection processes. Such inequality shares a robust link with individuals’ psychological responses, potentially weakening the positive predictive relationship typically associated with objective fairness. Consequently, this study posits that internet usage statistically moderates the relationship between perceived fairness in educational opportunities and social trust, manifesting as a negative interaction. Individuals characterized by high-intensity internet usage are more likely to be immersed in complex and conflicting information streams, frequently encountering discussions regarding the educational resource gap, the ossification of social class structures, or instances of educational injustice. Ultimately, this influx of negative information is hypothesized to buffer or weaken the positive association between perceived educational opportunity fairness and social trust. Accordingly, this paper proposes the following research hypotheses:

Hypothesis 5: The intensity of internet usage moderates the relationship between perceived educational opportunity fairness and social trust, manifesting as a negative interaction. Specifically, for individuals with a higher intensity of internet usage, the positive association between perceived educational opportunity fairness and social trust is significantly attenuated.

## 3 Materials and methods

### 3.1 Data source

This study utilizes household survey data from the 2023 wave of the CGSS. Established in 2003, the CGSS is China’s earliest nationwide, continuous academic survey project; it is representative, authoritative, and comprehensive in scope, covering political, economic, cultural, and social domains, and is widely employed in social science research. For this study, we selected the most recent CGSS dataset. To accurately and comprehensively reflect the empirical context, we excluded observations marked as “don’t know” or “refused to answer,” as well as outliers. The final sample comprises 5,175 valid observations, which provides sufficient statistical power for empirical analysis and inference. Given the extensive scope of this large-scale national survey data, the core psychological and sociological variables in this study-including perceived educational opportunity fairness, social trust, subjective well-being, and subjective social mobility expectations-were all operationalized using single-item measurements designed by the survey committee. It is worth emphasizing that, as one of China’s most authoritative and nationally representative continuous longitudinal surveys, the CGSS has long been recognized internationally for its academic rigor and data quality, and numerous high-level studies based on this data have been widely published in many prestigious SSCI journals.

Considering the specific context of this study, the use of single-item measurements is methodologically sound and applicable. Firstly, in nationwide longitudinal surveys with broad coverage and face-to-face interviews, methodologists widely acknowledge the strategic value of incorporating single-item measures. This approach not only significantly reduces the burden and potential survey fatigue of respondents and lowers administrative costs, but also maximizes the overall response and data completeness rates. In large-scale population surveillance or tracking surveys where testing space and time are constrained, single-item measurements offer a highly efficient and practical alternative, substantially mitigating the risk of response dropout caused by lengthy questionnaires (Burns and Crisp, 2026). Secondly, the social trust and subjective well-being explored in this study are coherent, well-defined, and essentially single-dimensional constructs, capturing an individual’s overall cognitive or emotional state. As Burns and Crisp demonstrated in validating the Brief Scales of Flourishing (BSF), single global items are sufficient to effectively capture individual differences in well-being continuum and exhibit satisfactory accuracy in classifying and predicting individual psychological states. Furthermore, although perceived fairness can encompass multiple micro-levels, this study treats “perceived fairness in educational opportunities” as a globally integrated, overarching construct, rather than an abstract and fragmented list of indicators. In the context of large-scale empirical research in China, researchers frequently and widely utilize a solitary survey item to characterize an individual’s general perception of fairness when examining macro-level social justice (Lu et al., 2024), which precisely aligns with the recommended conditions for single-item usage. Therefore, the measurement indicators employed in this study offer acceptable statistical power and academic rigor, providing valid empirical grounds for macro-level social psychological insights.

### 3.2 Variable selection

#### 3.2.1 Dependent variable

The dependent variable in this study is social trust. Drawing upon the work of previous scholars, it is measured using the following item from the CGSS project: “Generally speaking, do you agree that the vast majority of people in this society can be trusted?” Responses range from 1 to 5, where 1 = “strongly disagree,” 2 = “somewhat disagree,” 3 = “neutral,” 4 = “somewhat agree,” and 5 = “strongly agree.” A higher score reflects a higher level of social trust.

#### 3.2.2 Independent variable

The independent variable in this study is the perceived fairness of educational opportunities. It is operationalized via the CGSS project via the statement: “As long as children are sufficiently hardworking and intelligent, they will have equal opportunities for educational advancement.” Responses include “agree” and “disagree,” coded as follows: 1 = Agree and 2 = Disagree.

#### 3.2.3 Control variable

Drawing upon previous research and accounting for key covariates associated with social trust, we selected gender (Male = 1, Female = 2), age (survey year minus birth year), educational attainment (No formal education = 1; Private tutoring/Literacy classes = 2; Primary school = 3; Junior high school = 4; Vocational high school = 5; General high school = 6; Secondary specialized school = 7; Technical school = 8; Junior college [Adult higher education] = 9; Junior college [Regular higher education] = 10; Undergraduate [Adult higher education] = 11; Undergraduate [Regular higher education] = 12; Graduate studies or higher = 13), number of children (No children = 0, One or more children = 1), marital status (Unmarried = 0, Married = 1), and employment status (Farming = 0, Other employment = 1) as the control variables for this study.

#### 3.2.4 Mediating variable

The mediating variables in this study encompass the sense of social fairness, subjective well-being, and expectations of social class mobility. The sense of social fairness was measured using an item from the CGSS: “Overall, do you consider contemporary society to be fair or unfair?” Responses were recorded on a five-point scale-“completely unfair,” “somewhat unfair,” “neither fair nor unfair,” “somewhat fair,” and “very fair”-which were coded numerically from 1 to 5; a higher score reflects a stronger perception of social fairness. Subjective well-being is measured using an item from the CGSS: “Overall, would you say your life is happy?” Responses are recorded on a five-point scale-(Very unhappy = 1; Somewhat unhappy = 2; Average = 3; Somewhat happy = 4; Very happy = 5)-and are coded numerically from 1 to 5, with higher scores reflecting greater levels of personal happiness.

Expectations of social class mobility are captured using items from the CGSS: “In our society, some people occupy the upper strata, while others occupy the lower strata.” The ladder on this card [Card 5 is presented] should be viewed from top to bottom. The highest score, “10 represents the very top stratum, while the lowest score”; “1 represents the very bottom stratum.” Concretely speaking, “(a) Taking everything into account, at which level of society do you personally currently stand? (b) At which level do you believe you stood 10 years ago?” The discrepancy between the social class levels reported for 10 years ago and the present is utilized to operationalize the pattern of social class mobility. Internet usage is measured using an item from the CGSS: “Over the past year, What has been your usage of the following media? The Internet (accessing the web via mobile phones, computers, tablets, smart wearable devices, etc.)” A response indicating “no usage” is assigned a value of 0, while a response indicating “usage” is assigned a value of 1.

### 3.3 Statistical analysis and model building

This study employs Stata 17.0 for all statistical descriptions and analyses, utilizing an ordered Probit model to examine the relationship between perceived educational opportunity fairness and social trust. Firstly, we conduct descriptive statistical analyses of the key variables to characterize the basic features of the sample and the distribution of the variables. Secondly, we construct a baseline regression model to assess the association between perceived educational opportunity fairness and social trust. Building upon this, we perform robustness checks-by varying model specifications and variable operationalizations-to validate the reliability of the baseline results. Finally, we introduce interaction terms to further analyze how the association between perceived educational opportunity fairness and social trust may fluctuate conditional upon internet usage, thereby investigating how this predictive relationship varies across different information environments.

#### 3.3.1 Methodological basis

In empirical psychological research, both single-item measures and multi-item psychometric scales present distinct advantages and trade-offs (Gardner et al., 1998). No measurement protocol possesses universal applicability, and the choice between these approaches must be tailored to the specific design and evaluative purpose of the study (Wanous et al., 1997). On the one hand, while single-item measurement may not represent the theoretically optimal path under all circumstances, it effectively mitigates indicator redundancy and computational complexity, thereby aligning selected indicators more closely with the pragmatic constraints of field research (Allen et al., 2022). On the other hand, diagnosing and verifying measurement items through factor analysis helps ensure that the index design achieves a rigorous level of reliability and validity. This pre-test diagnostic approach largely overcomes the inherent limitations of single-item measures, thereby enhancing the scientific rigor and robustness of empirical conclusions (Watson and Wooden, 2021). Indeed, the application of single-item measures is highly established in the literature for assessing constructs such as job satisfaction (Wanous et al., 1997; Tong et al., 2025), subjective well-being (Raudenská, 2023), and generalized trust (Hoeppner et al., 2011). For these highly integrated or unidimensional variables, single-item measures demonstrate robust face validity and test-retest reliability, while substantially minimizing respondent fatigue (Matthews et al., 2022) in large-scale household surveys. Consequently, in large-scale national social surveys, single-item measurement has achieved widespread academic acceptance and empirical validation. This methodological precedent thoroughly rationalizes the adoption of single-item measures in the present study.

#### 3.3.2 Baseline regression model

Drawing upon the research of Cai Yingshu, the dependent variable in this paper is social trust ($T\,r\,u\,s\,t_{i}^{*}$). Taking values of 1, 2, 3, 4, and 5, it constitutes an ordered categorical variable (Cai et al., 2025); therefore, an Ordered Probit model is employed for estimation. The baseline empirical specification is formulated as follows:


$$T\,r\,u\,s\,t_{i}^{*}=\alpha_{0}+\alpha_{1}\,F\,a\,i\,r\,n\,e\,s\,s_{i}+\sum_{k=1}^{K}\beta_{k}\,C\,o\,n\,t\,r\,o\,l_{i\,k}+\varepsilon_{i}$$ (1)

In Equation 1, $T\,r\,u\,s\,t_{i}^{*}$ represents the unobservable latent variable of social trust for the *i*-th respondent; *Fairness*_*i*_ serves as the core independent variable, denoting the perceived fairness of educational opportunities. *Control*_*ik*_ denotes the control variables (including gender, age, years of schooling, presence of children, marital status, and employment status); α_0_ is the constant term; α_1_β_*k*_ are the coefficients to be estimated; and ε_*i*_ is the random disturbance term, which follows a standard normal distribution, ε∼iN(0,1).

Although ($T\,r\,u\,s\,t_{i}^{*}$) is an unobservable latent variable, it bears the following relationship to the observable ordered variable (*Trust_i_*):


T⁢r⁢u⁢s⁢ti={     1,Strongly⁢Disagree,if⁢T⁢r⁢u⁢s⁢ti*≤μ1     2,Disagree,if⁢μ1<T⁢r⁢u⁢s⁢ti*≤μ2     3,Neither⁢agree⁢nor⁢disagree,if⁢μ2<T⁢r⁢u⁢s⁢ti*≤μ3     4,Agree,if⁢μ3<T⁢r⁢u⁢s⁢ti*≤μ4     5,Strongly⁢agree,if⁢T⁢r⁢u⁢s⁢ti*>μ4 (2)

In Equation 2, (μ_1_ < μ_2_ < μ_3_ < μ_4_) are the threshold parameters (cut-off points) to be estimated.

From this, the probabilities of respondents selecting each level of social trust are obtained as follows:


$$\text{P}\left({\text{Trust}}_{\text{i}}=1|\text{X}\right)=\Phi \left(\mu_{1}-\alpha_{0}-\alpha_{1}{\text{Fairness}}_{\text{i}}-\sum \beta_{\text{k}}{\text{Control}}_{\text{ik}}\right)$$ (3)

$$\text{P}({\text{Trust}}_{\text{i}}=2|\text{X}=\Phi (\mu_{2}-\alpha_{0}-\alpha_{1}{\text{Fairness}}_{\text{i}}-$$

$$\sum \beta_{\text{k}}{\text{Control}}_{\text{ik}})-\Phi (\mu_{1}-\alpha_{0}-\alpha_{1}{\text{Fairness}}_{\text{i}}-\sum \beta_{\text{k}}{\text{Control}}_{\text{ik}})$$

$$\text{P}({\text{Trust}}_{\text{i}}=3|\text{X}=\Phi (\mu_{3}-\alpha_{0}-\alpha_{1}{\text{Fairness}}_{\text{i}}-$$

$$\sum \beta_{\text{k}}{\text{Control}}_{\text{ik}})-\Phi (\mu_{2}-\alpha_{0}-\alpha_{1}{\text{Fairness}}_{\text{i}}-\sum \beta_{\text{k}}{\text{Control}}_{\text{ik}})$$

$$\text{P}({\text{Trust}}_{\text{i}}=4|X=\Phi (\mu_{4}-\alpha_{0}-\alpha_{1}{\text{Fairness}}_{\text{i}}-$$

$$\sum \beta_{\text{k}}{\text{Control}}_{\text{ik}})-\Phi (\mu_{3}-\alpha_{0}-\alpha_{1}{\text{Fairness}}_{\text{i}}-\sum \beta_{\text{k}}{\text{Control}}_{\text{ik}})$$

$$\text{P}\left({\text{Trust}}_{\text{i}}=5|\text{X}\right)=1-\Phi (\mu_{4}-\alpha_{0}-\alpha_{1}{\text{Fairness}}_{\text{i}}-$$

$$\sum \beta_{\text{k}}{\text{Control}}_{\text{ik}})$$

In Equation 3, Φ(⋅) denotes the cumulative distribution function of the standard normal distribution. As with the binary Probit model, the parameters of the Ordered Probit model will be estimated using the maximum likelihood estimation method.

#### 3.3.3 Mediation effect model

To examine the mediating role of individual beliefs (social fairness, subjective well-being, and social class mobility), this paper draws upon the methodology of Cai et al. (2025) to construct the following recursive model:


$${\text{M}}_{\text{i}}=\gamma_{0}+\gamma_{1}\,{\text{Fairness}}_{\text{i}}+\sum \gamma_{\text{k}}\,{\text{Control}}_{\text{ik}}+\xi_{i\;}$$ (4)

$$T\,r\,u\,s\,t_{i}^{*}=\delta_{0}+\delta_{1}\,F\,a\,i\,r\,n\,e\,s\,s_{i}+\delta_{2}\,M_{i}+\sum \delta_{k}\,C\,o\,n\,t\,r\,o\,l_{i\,k}+\zeta_{i}$$ (5)

In Equations 4, 5, *M_i_* represents three mediating variables, respectively: social fairness (*SocialFair*_*i*_), happiness(*Happiness*_*i*_), and social class mobility (*Mobility*_*i*_).

#### 3.3.4 Moderating effect model

To examine the moderating role of internet usage, this study introduces an interaction term into Model (1) as follows:


$${\text{Trust}}_{\text{i}}^{*}=\&\theta_{0}+\theta_{1}\,{\text{Fairness}}_{\text{i}}+\theta_{2}\,{\text{Internet}}_{\text{i}}+$$ (6)

$$\theta_{3}\,\left(\mathrm{Fairnes}\,{\text{s}}_{\text{i}}\times {\text{Internet}}_{\text{i}}\right)+\sum \theta_{\text{k}}\,{\text{Control}}_{\text{ik}}+\upsilon_{\text{i}}$$

In Equation 6, the coefficient of the interaction term (θ_4_) represents the moderating effect of internet usage. To prevent multicollinearity, the variables constituting the interaction term were centered.

## 4 Results

### 4.1 Hamann single factor test

It should be acknowledged that because all core variables in this study were measured via self-assessment questionnaires, most constructs (e.g., trust, fairness, and happiness) relied on only one or two items, and the data were collected cross-sectionally at a single point in time. Consequently, the research methods and empirical conclusions might be susceptible to common method variance (CMV). To diagnose the severity of this potential issue and ensure the validity of subsequent regression analyses, we performed Harman’s single-factor test using principal component analysis (PCA). Unrotated factor analysis was conducted on all self-assessment measurement items. As indicated in Table 1, the variance explained by the first unrotated factor is 29.19%, which is well below the critical threshold of 50%. Furthermore, a distinct gap exists between the variance explained by the first factor (29.19%) and the second factor (14.03%), with no single factor dominating the total variance. These diagnostic results suggest that common method bias is not a serious concern in this study and that the variables exhibit sufficient discriminant validity for subsequent hypothesis testing.

**TABLE 1:** Hamann single factor test results.

Component	Eigenvalue	Variance explanation ratio (%)	Explanation ratio of cumulative variance (%)
1	2.3356	29.19	29.19
2	1.1224	14.03	43.22
3	1.0432	13.04	56.26
4	0.9694	12.12	68.38
5	0.9187	11.48	79.86
6	0.8037	10.05	89.91
7	0.5357	6.70	96.61
8	0.2712	3.39	100.00

### 4.2 Descriptive statistics

Descriptive statistics for each variable are presented in Table 2. The mean score for social trust is 3.53 (with a standard deviation of 0.94), reflecting an overall level that is slightly above average. The mean for perceived fairness of educational opportunities is 1.22 (with a standard deviation of 0.42), suggesting that a substantial majority of the sampled residents tend to hold a positive assessment concerning educational opportunity equity. The average age of the respondents is 50.33 years, the mean number of years of education is 8.37, and the internet usage rate is 45%. Furthermore, the mean for the sense of social fairness is 3.27, the mean for happiness is 3.90, and the mean for social class mobility is −0.67. The distribution of all variables is reasonable, satisfying the basic requirements for subsequent regression analysis.

**TABLE 2:** Main variables descriptive statistics.

Variables	Count	Mean	SD	Min	Max
Social trust	7420	3.53	0.94	1	5
Perceived educational opportunity fairness	7420	1.22	0.42	1	2
Gender	7420	1.54	0.50	1	2
Age	7420	50.33	16.57	18	94
Education	7420	8.37	4.55	0	18
Number of children	7420	1.41	0.49	1	2
Marital status	7420	1.79	0.41	1	2
Working or not	7420	1.59	0.49	1	2
Internet usage	7420	0.45	0.50	0	1
Social justice	7420	3.27	0.98	1	5
Sense of happiness	7420	3.90	0.81	1	5
Social mobility	7420	−0.67	1.99	−14	18

### 4.3 Baseline regression results

In this section, as shown in Table 3, we constructed two models. In Model (1), the coefficient for perceived educational opportunity fairness was 0.216 (*p* < 0.01), reflecting that-without controlling for other variables-perceived educational opportunity fairness serves as a significant positive predictor of perceived educational opportunity fairness social trust. In Model (2), after controlling for variables such as gender, age, years of schooling, marital status, and employment status, the coefficient decreased to 0.163 but remained highly significant. This suggests that the positive statistical association between perceived educational opportunity fairness and social trust persists robustly perceived educational opportunity fairness even after controlling for individual characteristics.

**TABLE 3:** Baseline regression analysis of perceived educational opportunity fairness social trust.

Model variables	(1) Without control variables	(2) With control variables
Perceived educational opportunity fairness	0.216***	0.163***
	(0.043)	(0.044)
Gender		−0.087***
		(0.031)
Age		0.006***
		(0.001)
Years of education		0.017***
		(0.004)
Presence of children		−0.083*
		(0.044)
Marital status		0.054
		(0.034)
Working or not		−0.142***
		(0.041)
Observations	5175	5175
Pseudo R^2^	0.002	0.009
*P*-value	0.000	0.000

*p < 0.1,

***p < 0.01. Standard errors in parentheses.

### 4.4 Robustness check results

To further verify the robustness of the results, several tests were conducted, and the findings are summarized in Table 4. First, when using the Ordered Logit model for estimation, the coefficient for perceived educational opportunity fairness is 0.283 and is significantly positive. Second, linear regression using Ordinary Least Squares (OLS) yields a coefficient of 0.141, which is also significant. Finally, after introducing depression into the model as an additional control variable to account for potential psychological confounding, the coefficient for perceived educational opportunity fairness remains significant at 0.151. These results consistently suggest that the positive statistical association between perceived educational opportunity fairness and social trust exhibits high empirical stability across varying model specifications.

**TABLE 4:** Robustness test analysis of perceived educational opportunity fairness social trust.

Model variables	(1) Ordered Logit	(2) OLS	(3) Additional controls
Perceived educational opportunity fairness	0.283***	0.141***	0.151***
	(0.076)	(0.042)	(0.045)
Gender	−0.158***	−0.068**	−0.080***
	(0.054)	(0.029)	(0.031)
Age	0.011***	0.005***	0.006***
	(0.002)	(0.001)	(0.001)
Years of education	0.030***	0.017***	0.015***
	(0.007)	(0.004)	(0.004)
Presence of children	−0.149*	−0.076*	−0.080*
	(0.079)	(0.042)	(0.044)
Marital status	0.104*	0.049	0.050
	(0.059)	(0.032)	(0.034)
Working or not	−0.244***	−0.132***	−0.146***
	(0.073)	(0.038)	(0.041)
Depression			0.055***
			(0.014)
Observations	5175	5175	5175
Pseudo R^2^	0.009	0.020	0.010
R-squared		
*P*-value	0.000	0.000	0.000

*p < 0.1,

**p < 0.05,

***p < 0.01. Standard errors in parentheses.

### 4.5 Mediation analysis results

To elucidate the underlying mechanisms through which perceived educational opportunity fairness correlates with social trust, this study constructs a mediation effects model, the results of which are presented in Table 5. Model (2) indicates that perceived educational opportunity fairness (PEOF) exhibits a significant positive predictive relationship with the sense of social fairness (coefficient = 0.266). In Model (3)-after controlling for the sense of social fairness-the coefficient for perceived educational opportunity fairness decreases to 0.079, while the coefficient for the sense of social fairness remains significantly positive (0.366); this suggests that the sense of social fairness functions as a partial mediator within this statistical framework. Similarly, both subjective well-being (Models 4–5) and expectations of social class mobility (EoSCM, Models 6–7) exhibit significant indirect pathways. Consequently, these findings suggest that perceived educational opportunity fairness is not only directly associated with social trust but it also shares indirect positive links that are systematically channeled through heightened levels of the sense of social fairness, subjective well-being, and expectations of social class mobility.

**TABLE 5:** Mediating analysis of perceived educational opportunity fairness and social trust.

Models	(1)	(2)	(3)	(4)	(5)	(6)	(7)
Variables	Baseline	Social fairness	Y-social trust	Subjective well-being	Y-social trust	EoSCM	Y-social trust
PEOF	0.163***	0.266***	0.079*	0.220***	0.128***	0.117***	0.158***
	(0.044)	(0.044)	(0.045)	(0.045)	(0.045)	(0.044)	(0.044)
Social justice			0.366***				
			(0.015)				
Sense of happiness					0.240***		
					(0.018)		
Social mobility							0.020**
							(0.009)
Control variable	Yes	Yes	Yes	Yes	Yes	Yes	Yes
Observations	5175	5175	5175	5175	5175	5175	5175
Pseudo R^2^	0.009	0.006	0.051	0.010	0.022	0.034	0.009
*P*-value	0.00	0.00	0.00	0.00	0.00	0.00	0.00

*p < 0.1,

**p < 0.05,

***p < 0.01. Standard errors in parentheses.

### 4.6 Results of moderating effect analysis

This paper further examines the moderating role of internet use in the relationship between perceived educational opportunity fairness and social trust; the results are presented in Table 6. In Model (1), the coefficient for the interaction term between perceived educational opportunity fairness and internet use is −0.374 (*p* < 0.05). This result indicates that the intensity of internet use exerts a significant negative moderating effect on this relationship-specifically, internet use attenuates the positive impact of perceived educational opportunity fairness on social trust. Internet use not only directly influences social trust but also disrupts the “equity-trust” causal pathway by altering individuals’ information exposure environments. This finding strongly supports Hypothesis 5, which posits that internet use plays a significant negative moderating role in the positive relationship between perceived educational opportunity fairness and social trust.

**TABLE 6:** Moderating analysis of perceived educational opportunity fairness and social trust.

Model variables	(1) Internet usage
Perceived educational opportunity fairness	0.502***
	(0.146)
Internet usage	0.609**
	(0.300)
Perceived educational opportunity fairness # internet usage	−0.374**
	(0.153)
Control variable	Yes
Observations	5175
Pseudo R^2^	0.010
*P*-value	0.000

**p < 0.05,

***p < 0.01. Standard errors in parentheses.

## 5 Conclusion

This study examines the issue of social trust among Chinese residents within the analytical framework of perceived educational opportunity fairness. Utilizing data from the 2023 CGSS, it analyzes statistical association between perceived educational opportunity fairness and social trust, while exploring the underlying pathways through which this predictive relationship operates. The main findings are as follows: Firstly, perceived educational opportunity fairness exhibits a significant positive association with levels of social trust. Whether controlling for individual characteristics-such as gender, age, years of schooling, marital status, and employment status-or employing robustness checks via models such as Ordered Logit and OLS, perceived educational opportunity fairness consistently demonstrates a stable predictive relationship with social trust. This indicates that perceived educational opportunity fairness relates systematically to the variation in social trust, and this statistical link remains consistent across various estimation methods. Secondly, perceived educational opportunity fairness shares an indirect relationship with social trust, which is systematically channeled through the sense of social fairness, subjective well-being, and expectations of social class mobility; the relevant variables all demonstrate significant effects within the models. Specifically, perceived educational opportunity fairness relates positively to residents’ perceptions of the fairness of social institutions, which systematically corresponds to higher social trust. Concurrently, perceived educational opportunity fairness is positively tied to residents’ subjective well-being, which in turn serves as a robust predictor of social trust. Furthermore, perceived educational opportunity fairness aligns with higher expectations of upward social class mobility, thereby corresponding to higher levels of social trust. These results reveal a multi-path mediation mechanism whereby perceived educational opportunity fairness connects with social trust, reflecting the combined interplay of cognitive perceptions, emotional states, and developmental expectations. Thirdly, internet usage statistically moderates the relationship between perceived educational opportunity fairness and social trust, manifesting as a negative interaction. Model analyses indicate that the coefficient for the interaction term between perceived educational opportunity fairness and internet usage is negative, suggesting that for individuals with a higher intensity of internet usage, the positive association between perceived educational opportunity fairness and social trust is significantly attenuated. This finding suggests that, within a digitized environment, the social perceptions regarding educational equity and their alignment with trust are conditional upon the online information landscape. Fourthly, both the direct and indirect associations of perceived educational opportunity fairness with social trust demonstrate empirical stability. Even after employing different estimation methods and introducing additional control variables, the positive predictive relationship remains statistically significant, thereby affirming the robustness of these findings.

Overall, the findings of this study demonstrate consistency across various mechanistic pathways, indicating that perceived educational opportunity fairness correlates with social trust through three distinct interrelated channels: cognitive (sense of social fairness), affective (subjective well-being), and aspirational (expectations of social class mobility). Concurrently, variations in the intensity of these mechanisms suggest that the institutional-cognitive pathway (sense of fairness) constitutes the core mechanism, while the affective and developmental-aspirational pathways serve as complementary explanatory factors. Furthermore, internet usage acts as a contextual variable that significantly shapes the boundaries of this relationship, thereby underscoring the pronounced environmental contingency inherent in the statistical variation of social trust. To deeply explore the negative moderating effect of high-intensity internet use, an explanation can be derived by integrating negativity bias theory and upward social comparison theory. Negativity bias theory weakens the effective conversion of positive cognitive cues in reality through high-frequency algorithmic bombardment, while upward social comparison theory induces status anxiety and relative deprivation by raising the psychological benchmark of comparison.

## 6 Discussion

### 6.1 Theoretical contributions

Negativity bias theory posits that negative events, experiences, or information exert a significantly greater impact on individual judgments and behaviors than equivalent positive inputs (Rozin and Royzman, 2001). In communication science and media studies, this phenomenon typically manifests as the dominant prevalence of negative communication across social media and digital networks, with negative content eliciting much stronger emotional and behavioral responses from users than positive information. Under high-intensity digital consumption, users are more frequently exposed to problematized, conflict-ridden, and negatively oriented content, as algorithmic recommendation engines tend to amplify negative news such as social injustice and educational anxieties. From a psychological perspective, this high-frequency bombardment of negative information alienates the entire online landscape into a “poor context” with an overall negative expected return, leading negative outcomes to be severely overweighted at the level of cognitive parameters. As reflected by some scholars have analyzed, perceived negativity bias is significantly and positively related to feelings of accountability, while inversely co-varying with perceived social support (Zoonen et al., 2024). Communication visibility disproportionately amplifies the psychological weight of negative comments and obstacles, rendering the digital consumption space an “unsafe psychological environment” devoid of social support and filled with high risks of potential criticism. This demonstrates that the negativity bias within the internet context functions as a cognitive attenuator that weakens the systemic translation of positive micro-level cognitive cues (such as perceived educational fairness) into macro-level institutional trust (such as social trust), thereby diluting the positive predictive role that objective fairness experiences should play.

In addition, high-intensity digital consumption severely impacts individuals’ self-evaluation from the perspective of reference benchmarks while distorting the quality of information acquisition. Based on social comparison theory (Festinger, 1954), people often unconsciously compare themselves with others, and high-intensity online digital consumption forces individuals into high-frequency “upward social comparison”-the process of choosing to compare oneself with people who are superior to them in certain aspects. Studies have found that a higher degree of upward social comparison increases the probability of experiencing higher levels of social anxiety (Xu and Li, 2024). Upward social comparison directly triggers a profound sense of relative deprivation; that is, by comparing themselves with a tiny minority of top elites on the internet, individuals subjectively believe that they should possess, but actually lack, those enviable resources. Even if an individual has achieved upward class mobility through education in reality and subjectively believes that the “educational selection process in reality is fair,” when they are intensely immersed in the network, those amplified and deliberately exaggerated “wealthy second-generation” or “elite classes” on the internet will still induce a strong sense of relative deprivation and status anxiety through high-frequency upward social comparison.

### 6.2 Practical implications

This finding suggests to policymakers that, in striving for the equitable allocation of educational resources, attention must extend beyond mere objective inputs to ensure the transparency and fairness of distribution procedures, thereby mitigating the potential confounding from non-effort factors (such as household registration status and family background). Based on the conclusions of this study, and with the aim of further bolstering social trust, mechanisms safeguarding equal educational opportunities should be refined. It is essential to strictly implement the requirements for educational equity stipulated in the Education Law of the People’s Republic of China, ensuring the fair distribution of various educational resources across urban and rural areas, different regions, and diverse social groups. Specifically targeting rural populations, low-income families, and vulnerable groups, efforts should focus on expanding investment in basic education, optimizing the allocation of teaching staff, and refining systems for grants and scholarships. These measures aim to align students’ actual experiences with equal opportunities regarding educational resources, curriculum content, and school environments, with a particular emphasis on enhancing their subjective sense of fairness. Furthermore, in critical stages such as student admissions, examinations, and recommendations for postgraduate study, more open and transparent oversight mechanisms should be established to minimize the disruptive links of non-effort factors-such as personal connections and family background-in the distribution of educational opportunities. At the societal level, a robust social security system should be established to alleviate the fear among citizens of downward social mobility. When individuals perceive that they can still secure a decent livelihood and adequate social protection-even if they do not reach the very top tier of the educational selection process-their subjective well-being is sustained, thereby serving as a psychological buffer that protects social trust from fluctuations driven by anxiety.

In addition to macro-level educational policy adjustments, this study also offers important implications for micro-level psychosocial interventions. One the one hand, school mental health centers and community health service institutions should introduce targeted cognitive media literacy courses to help individuals identify the false top-tier reference frameworks amplified by algorithms. These courses should guide students and young people to realize that online myths of sudden wealth and elite lifestyles are merely highlight reels carefully curated by algorithms and users, thereby reducing the status anxiety brought by such false reference systems. On the other hand, close attention must be paid to the perceived fairness of educational opportunities. This is crucial to prevent the negative effects of educational scarcity and perceived social injustice from superimposing on social trust and subsequently undermining residents’ subjective well-being. For regions characterized by underdeveloped education and inequitable resource distribution, higher social attention and differentiated, collaborative resource allocation should be highly warranted. Specifically, the further expansion of educational inequality can be systematically prevented by establishing a dynamic balance between educational development and psychosocial service systems, deepening structural reforms in resource allocation policies, and precisely targeting the needs of multidimensional heterogeneous groups. Ultimately, this micro-level preservation of individuals’ perceived fairness regarding educational opportunities can be effectively translated into social trust at the macro level.

### 6.3 Limitations and future research directions

Several potential limitations of this study warrant explicit acknowledgment. Firstly, a primary methodological constraint arises from the measurement of key variables, which relies exclusively on single-item indicators from the CGSS questionnaire. While single-item measures are widely adopted in large-scale social surveys for their efficiency and high face validity, they inherently limit our ability to comprehensively capture multi-dimensional facets of these social perceptions. Secondly, attributable to the data nature and specific characteristics of the CGSS dataset, this research relies entirely on a cross-sectional design. Consequently, while our ordered Probit and mediation models provide robust evidence of statistical associations, they are inherently constrained in verifying strict chronological causality. Specifically, the possibility of reverse causality cannot be entirely ruled out within this snapshot framework. For instance, while individuals with a stronger perceived educational opportunity fairness may exhibit higher social trust, it is equally plausible that individuals possessing inherently higher social trust are more inclined to evaluate social institutions and educational resources allocation as fair. Therefore, future research utilizing multi-item instruments, as well as longitudinal or panel datasets, will be essential to enhance measurement precision, track dynamic shifts over time, and further disentangle these causal directions. Moreover, our future related research will extend the quasi-social experiments and multi-social field research, and reproduce it with more detailed psychological clinical scales, and then further analyze the causal relationship and specific action pathway between perceived educational opportunity fairness and social trust.

## Data Availability

The original contributions presented in this study are included in the article/supplementary material, further inquiries can be directed to the corresponding author.

## References

[B1] Akaeda N. (2022). Social trust and well-being inequality according to social stratification. *Res. Soc. Stratif. Mobil.* 82:100733. 10.1016/j.rssm.2022.100733

[B2] Akokuwebe M. E. Idemudia E. S. (2022). A comparative cross-sectional study of the prevalence and determinants of health insurance coverage in Nigeria and South Africa: A multi-country analysis of demographic health surveys. *Int. J. Environ. Res. Public Health* 19:1766. 10.3390/ijerph19031766 35162789 PMC8835528

[B3] Akokuwebe M. E. Likoko S. Miles-Timotheus S. Mabala M. (2025). “Gender dimensions of quality of life”: The determinants of life satisfaction among South Africans residing in the Gauteng province—Using a multilevel psychodemographic analysis of the GCRO’s quality of life survey (2009–2024). *PLoS One* 20:e0332626. 10.1371/journal.pone.0332626 40953112 PMC12435731

[B4] Albanesi C. Cicognani E. Zani B. (2007). Sense of community, civic engagement and social well-being in Italian adolescents. *J. Community Appl. Soc. Psychol.* 17 387–406. 10.1002/casp.903

[B5] Albertazzi A. Lown P. Mengel F. (2025). Income inequality and social trust. *Econ. Lett.* 257:112675. 10.1016/j.econlet.2025.112675

[B6] Allan W. Jacquelynne S. E. (2000). Expectancy–value theory of achievement motivation. *Contemp. Educ. Psychol.* 25 68–81. 10.1006/ceps.1999.1015 10620382

[B7] Allen M. S. Iliescu D. Greiff S. (2022). Single item measures in psychological science: A call to action. *Eur. J. Psychol. Assess.* 38 1–5. 10.1027/1015-5759/a000699

[B8] Bourdieu P. Passeron J. C. (1990). *Reproduction in Education, Society and Culture*, 2nd Edn. London: SAGE Publications.

[B9] Breen R. Jonsson J. O. (2005). Inequality of opportunity in comparative perspective: Recent research on educational attainment and social mobility. *Annu. Rev. Sociol.* 31 223–243. 10.1146/annurev.soc.31.041304.122232

[B10] Burns R. A. Crisp D. A. (2026). The development of single-item measures of flourishing: The Brief Scales of Flourishing (BS-F). *J. Posit. Psychol.* 21 536–551. 10.1080/17439760.2025.2502486

[B11] Cai Y. Zhang B. Zou X. Li H. J. (2025). Risk perceptions and personal beliefs on climate adaptation behavior of large grain farmers. *Chinese J. Agric. Resour Regional Plann.* 46 13–26.

[B12] Cary W. (2021). Education and social trust in global perspective. *Sociol. Perspect.* 64 1166–1186. 10.1177/0731121421990045

[B13] Checchi D. Peragine V. (2010). Inequality of opportunity in Italy. *J. Econ. Inequal.* 8 429–450. 10.1007/s10888-009-9118-3

[B14] Chen D. (2006). A study on relation between fairness and efficiency in the development of higher education. *Soc. Sci. Yunnan* 2006 44–47.

[B15] Chen X. Xiang K. (2021). The development and revelation of the Communist Party of China’s centennial anti-poverty cause. *J. Huazhong Agric. Univ.* 155 5–13. 10.13300/j.cnki.hnwkxb.2021.05.002

[B16] Cheng X. (2020). Malaise effect or virtuous effect? The dynamics of internet use and political trust in China. *Int. J. Commun.* 14 1995–2016.

[B17] Cho S. Y. (2016). Does gender equality promote social trust? An empirical analysis. *World Dev.* 88 175–187. 10.1016/j.worlddev.2016.07.019

[B18] Coleman J. S. (1975). Equal educational opportunity: A definition. *Oxf. Rev. Educ.* 1 25–29. 10.1080/0305498750010104

[B19] Day M. V. Fiske S. T. (2017). Movin’ on up? How perceptions of social mobility affect our willingness to defend the system. *Soc. Psychol. Personal. Sci.* 8 267–274. 10.1177/1948550616678454 30766651 PMC6371776

[B20] Diener E. (1984). Subjective well-being. *Psychol. Bull.* 95 542–575. 10.1007/978-90-481-2350-6_2 6399758

[B21] Dworkin R. (1981). What is equality? part 1: Equality of welfare. *Philos. Public Aff.* 10 185–246. 10.4324/9781315199795-6

[B22] Fershtman C. Gneezy U. List J. A. (2012). Equity aversion: Social norms and the desire to be ahead. *Am. Econ. J. Microecon.* 4 131–144. 10.1257/mic.4.4.131

[B23] Festinger L. (1954). A theory of social comparison processes. *Hum. Relat.* 7 117–140. 10.1177/001872675400700202

[B24] Flores F. Solomon R. C. (1998). Creating trust. *Bus. Ethics Q.* 8 205–232. 10.2307/3857326

[B25] Gao S. Zhao J. (2022). The influence of perception of social equality and social trust on subjective well-being among rural Chinese people: The moderator role of education. *Front. Psychol.* 12:731982. 10.3389/fpsyg.2021.731982 35046863 PMC8762249

[B26] Gardner D. Cummings L. Dunham R. (1998). Single-item versus multiple-item measurement scales: An empirical comparison. *Educ. Psychol. Meas.* 58 898–915. 10.1177/0013164498058006003

[B27] Golley J. Kong S. T. (2018). Inequality of opportunity in China’s educational outcomes. *China Econ. Rev.* 51 116–128. 10.1016/j.chieco.2016.07.002

[B28] Guillen L. Zeichner K. (2018). A university-community partnership in teacher education from the perspectives of community-based teacher educators. *J. Teach. Educ.* 69 140–153. 10.1177/0022487117751133

[B29] He Q. Tong H. Liu J.-B. (2022). How does inequality affect the residents’ subjective well-being: Inequality of opportunity and inequality of effort. *Front. Psychol.* 13:843854. 10.3389/fpsyg.2022.843854 35465572 PMC9019070

[B30] Hoeppner B. B. Kelly J. F. Urbanoski K. A. Slaymaker V. (2011). Comparative utility of a single-item versus multiple-item measure of self-efficacy in predicting relapse among young adults. *J. Subst. Abuse Treat.* 41 305–312. 10.1016/j.jsat.2011.04.005 21700411 PMC3315352

[B31] Hou B. Liu Q. Wang Z. Hou J. Chen S. (2023). The intermediary mechanism of social fairness perceptions between social capital and farmers’ political participation: Empirical research based on masking and mediating effects. *Front. Psychol.* 13:1021313. 10.3389/fpsyg.2022.1021313 36698580 PMC9868247

[B32] Huang J. Maassen, van den Brink H. Groot W. (2009). A meta-analysis of the effect of education on social capital. *Econ. Educ. Rev.* 28 454–464. 10.1016/j.econedurev.2008.03.004

[B33] Ishimaru A. M. Rajendran A. Nolan C. M. Bang M. (2018). Community design circles: Co-designing justice and wellbeing in family-community-research partnerships. *J. Fam. Divers. Educ.* 3 38–63. 10.53956/jfde.2018.133

[B34] Kanitsar G. (2022). The inequality-trust nexus revisited: At what level of aggregation does income inequality matter for social trust? *Soc. Indic. Res.* 163 171–195. 10.1007/s11205-022-02894-w

[B35] Kus L. Ward C. Liu J. (2014). Interethnic factors and subjective well-being. *Polit. Psychol.* 35 703–719. 10.1111/pops.12038

[B36] Lamertz K. (2002). The social construction of fairness: Social influence and sense making in organizations. *J. Organiz. Behav.* 23 19–37. 10.1002/job.128

[B37] Li N. He M. (2022). Social security satisfaction and people’s subjective wellbeing in China: The serial mediation effect of social fairness and social trust. *Front. Psychol.* 13:855530. 10.3389/fpsyg.2022.855530 35444600 PMC9014885

[B38] Liu Y. Wei H. (2025). Will alleviating energy poverty enhance social trust in China? An approach based on dual machine learning modeling. *Energy Econ.* 147:108560. 10.1016/j.eneco.2025.108560

[B39] Lu H. Tong P. Zhu R. (2020). Longitudinal evidence on social trust and happiness in China: Causal effects and mechanisms. *J. Happiness Stud.* 21 1841–1858. 10.1007/s10902-019-00159-x

[B40] Lu H. Zeng K. Yang L. (2024). Perceptions of social mobility and political trust in China: The mediating role of perceived fairness. *Soc. Just. Res.* 37 382–405. 10.1007/s11211-024-00442-0

[B41] Matthews R. A. Pineault L. Hong Y. H. (2022). Normalizing the use of single-item measures: Validation of the single-item compendium for organizational psychology. *J. Bus. Psychol.* 37 639–673. 10.1007/s10869-022-09813-3

[B42] Mayhew M. J. Fernández S. D. (2007). Pedagogical practices that contribute to social justice outcomes. *Rev. High. Educ. J. Assoc. Study High. Educ.* 31 55–80. 10.1353/rhe.2007.0055

[B43] Melita D. Gobel M. Matamoros-Lima J. Rodríguez-Bailón R. Willis G. (2024). *Perceived Downward Mobility Increases Status Anxiety and Reduces Subjective Wellbeing.* Charlottesville, VA: OSF Preprints, 10.31219/osf.io/kjdsb

[B44] Merten L. Niedringhaus J. (2025). When perception shapes reality: Effects of perceived income inequality and social mobility on affective polarization. *J. Econ. Inequal.* 23 327–347. 10.1007/s10888-024-09641-w

[B45] Österman M. (2021). Can we trust education for fostering trust? quasi-experimental evidence on the effect of education and tracking on social trust. *Soc. Indic. Res.* 154 211–233. 10.1007/s11205-020-02529-y

[B46] Rahn W. M. Transue J. E. (1998). Social trust and value change: The decline of social capital in American youth, 1976–1995. *Polit. Psychol.* 19 545–565. 10.1111/0162-895X.00117

[B47] Raudenská P. (2023). Single-item measures of happiness and life satisfaction: The issue of cross-country invariance of popular general well-being measures. *Humanit. Soc. Sci. Commun.* 10:861. 10.1057/s41599-023-02299-1

[B48] Rozin P. Royzman E. B. (2001). Negativity bias, negativity dominance, and contagion. *Pers. Soc. Psychol. Rev.* 5 296–320. 10.1207/S15327957PSPR0504_2 42559194

[B49] Shang S. Y. Su Y. J. (2020). Association between moral cognition, moral emotion and prosocial behavior: Evidence from meta-analysis (in Chinese). *Chin. Sci. Bull.* 65 2021–2031. 10.1360/TB-2019-0676

[B50] Stanley J. Hensher D. A. Stanley J. Currie G. Greene W. H. Vella-Brodrick D. (2011). Social exclusion and the value of mobility. *J. Transp. Econ. Policy* 45 197–222. 10.3828/jtep.2011.45.2.197

[B51] Sui X. Zhao B. Na D. Liu J. Zhang Q. (2024). The relationship between physical exercise and sense of social fairness among college students: The chain-mediated role of perceived social support and life satisfaction. *Front. Psychol.* 15:1430492. 10.3389/fpsyg.2024.1430492 39228874 PMC11368798

[B52] Tong L. K. Li Y. Y. Liu Y. B. Zheng M. R. Fu G. L. Au M. L. (2025). Validation of the short index of job satisfaction in Chinese nurses: Classical test theory and item response theory. *Int. J. Nurs. Stud. Adv.* 8:100321. 10.1016/j.ijnsa.2025.100321 40276212 PMC12018056

[B53] Tyler T. R. (2006). *Why People Obey the Law.* Princeton, NJ: Princeton University Press.

[B54] Van de Gaer D. Ramos X. (2020). Measurement of inequality of opportunity based on counterfactuals. *Soc. Choice Welf.* 55 595–627. 10.1007/s00355-020-01261-3

[B55] Van Zoonen W. van der Meer T. Sivunen A. (2024). Expectation dissonance: The role of perceived negativity bias in enterprise social media in explaining accountability and support. *Inf. Technol. People* 37 196–215. 10.1108/ITP-05-2023-0502

[B56] Vetter A. Faircloth B. S. Hewitt K. K. Gonzalez L. M. He Y. Rock M. L. (2022). Equity and social justice in research practice partnerships in the United States. *Rev. Educ. Res.* 92 829–866. 10.3102/00346543211070048

[B57] Wanous J. P. Reichers A. E. Hudy M. J. (1997). Overall job satisfaction: How good are single-item measures? *J. Appl. Psychol.* 82 247–252. 10.1037/0021-9010.82.2.247 9109282

[B58] Watson N. Wooden M. (2021). The household, income and labour dynamics in Australia (HILDA) survey. *Jahrb. Natl. Stat.* 241 131–141. 10.1515/jbnst-2020-0029 38553031

[B59] Willis G. B. Uchida Y. García-Castro J. D. Takemura K. (2024). High relational mobility is associated with perceiving more economic inequality in everyday life. *Asian J. Soc. Psychol.* 27 348–359. 10.1111/ajsp.12597

[B60] Xu L. Li L. (2024). Upward social comparison and social anxiety among Chinese college students: A chain-mediation model of relative deprivation and rumination. *Front. Psychol.* 15:1430539. 10.3389/fpsyg.2024.1430539 39081379 PMC11286571

[B61] Yang J. Zhang L. Zhu J. (2025). Educational inequality and residents’ social trust in China: The role of inequality of opportunity. *Asian J. Soc. Sci.* 53:100202. 10.1016/j.ajss.2025.100202

[B62] You J. (2012). Social trust: Fairness matters more than homogeneity. *Polit. Psychol.* 33 701–721. 10.1111/j.1467-9221.2012.00893.x

[B63] You Y. Yu S. Xiao S. (2022). Once accessing the internet, less trusting of local officials? *Society* 59 723–734. 10.1007/s12115-022-00758-0

[B64] Yu G. (2026). Equity in mental health education: Possibility, necessity, and feasibility. *J. Natl. Acad. Educ. Adm.* 2026 9–22.

[B65] Zeichner K. Bowman M. Guillén L. Napolitan K. (2016). Engaging and working in solidarity with local communities in preparing teachers of their children. *J. Teach. Educ.* 67 277–290. 10.1177/0022487116660623

